# Epidemiology of non-unions following foot and ankle fractures: a nationwide analysis from 2014 to 2023

**DOI:** 10.1186/s12891-026-09755-4

**Published:** 2026-03-24

**Authors:** Annette Eidmann, Philipp Herrmann, Felix Hochberger, Sebastian Frischholz, Manuel Weißenberger, Maximilian Rudert, Ioannis Stratos

**Affiliations:** 1 Department of Orthopaedic Surgery, Julius-Maximilians Universität Wuerzburg, Koenig-Ludwig-Haus Brettreichstrasse 11, Wuerzburg, 97074 Germany; 2 OCW Orthopädie Chirurgie Wuerzburg, Oeggstraße 3, Wuerzburg, 97070 Germany

**Keywords:** Epidemiological analysis, Foot and Ankle Fractures, Delayed union, Non-union, Age distribution

## Abstract

**Background:**

Non-unions are a common complication following fractures. Although fractures of the foot and ankle are frequent, reliable data on the incidence and epidemiology of non-unions in these regions are lacking.

**Methods:**

A nationwide retrospective analysis was conducted using inpatient data from the German Federal Statistical Office between 2014 and 2023. Fractures and non-unions of the foot and ankle were identified via ICD-10 coding, with two cohorts defined: fracture cases and non-union cases. Data were stratified by age and sex, and all co-coded secondary diagnoses were extracted and aggregated to identify the most frequent comorbidities. Temporal trends were assessed using linear regression, while age distributions were modelled with two-component Gaussian mixture models to capture distinct etiological subpopulations. Data processing, statistical analyses, and visualization were performed in R (Version 4.3) using Tidyverse packages.

**Results:**

Between 2014 and 2023, 992,360 foot and ankle fractures and 20,268 non-unions of the foot and ankle were treated in hospitalized patients in German hospitals. Women were more frequently affected by both fractures and non-unions than men. The age distribution was bimodal, with peaks at 18–29 and 50–59 years in men, and at 50–79 years in women. The overall non-union rate was 2.0% and showed a declining trend over time. Age- and sex-specific differences were observed, with the highest non-union rate in women aged 40–49 years (3.2%). Comorbidities including obesity, allergies, depressive episodes, asthma, and sleep disorders were more prevalent in patients with non-unions compared to those with fractures.

**Conclusions:**

Non-unions following fractures of the foot and ankle remain rare and have declined steadily over the past decade. Age- and sex-specific differences underscore the need for targeted prevention strategies.

## Introduction

Bone fractures are common, with a lifetime prevalence of more than 40% at the age of 55 [1]. Despite substantial advances in the management of fractures and associated soft-tissue injuries, non-unions remain among the most frequent complications. They present significant challenges not only for treating surgeons but also for patients, in whom they impair physical integrity, well-being, and, at times, mental health [2]. Moreover, non-unions impose considerable economic burdens, both through prolonged treatment and indirectly through productivity loss [3–5].

Fractures of the foot and ankle, in particular, are common fractures. With an incidence of 49 per 100,000, lateral malleolar fractures represent the seventh most common fracture in Germany [6]. These injuries occur across all age groups, affecting both young, physically active individuals and older patients, in whom they often present as insufficiency fractures. In the literature, the rate of non-unions after fractures is given as 5–10%, but in some cases as high as 10–15% [7, 8]. However, these rates are general and do not refer specifically to fractures of the foot and ankle. Anatomical features of the so-called “last meadows” of the ankle and, in particular, the foot have led to the clinical impression that these sites may be especially prone to non-union. Walter et al. report an incidence of non-unions of the foot and ankle of 2.9/100,000 inhabitants in Germany [5]. In another, population-based study, the rate of non-unions was reported to be only 1.9% per fracture, and in the foot and ankle joint only around 1% [9]. However, this study included just 471 cases from the foot and ankle. Data from a larger population specifically examining the prevalence of non-unions after fractures of the foot and ankle are lacking. Thus, there is also a lack of data on the age and gender distribution as well as the temporal development of non-unions of the foot and ankle.

The objective of the present study was therefore to analyse the rate of non-unions following fractures of the foot and ankle, with particular attention to age, sex, and temporal patterns. The hypotheses to be tested are that non-unions of the foot and ankle occur at a rate lower than the 10% reported in the literature and that there is an age- and gender-specific distribution pattern.

## Materials and methods

### Study design

For the present study, data from the German Federal Statistical Office were analysed, comprising all fully hospitalized patients treated between 2014 and 2023. Diagnoses were coded according to the ICD-10 classification. Two cohorts were defined: the non-union cohort, including diagnoses M84.17 (non-union of fractures of the foot and ankle) and M84.17 (delayed union of fractures of the foot and ankle), and the fracture cohort. Non-unions and delayed unions were considered as a single group (“non-unions”) at all stages of the analysis, since the distinction depends on the quality of coding rather than on a precise medical definition. As ankle fractures are subsumed under lower-leg fractures, the relevant subcategories had to be queried separately (S82.3: fracture of the lower end of the tibia; S82.5: fracture of the medial malleolus; S82.6: fracture of the lateral malleolus; S82.8: fracture of other parts of the lower leg, including bi- and trimalleolar fractures). Fractures of the foot were subsumed under code S92.

All datasets were stratified by sex and age. Furthermore, for each of the primary diagnoses, all co-coded secondary diagnoses were retrieved. Secondary diagnoses were aggregated separately for the non-union and fracture cohorts across the entire study period. The ten most frequent secondary diagnoses in each cohort were identified, and their prevalence was calculated as the ratio of patients with the respective secondary diagnosis to the total number of patients with fracture or non-union. Secondary diagnoses with direct etiological or procedural links to the primary diagnosis (e.g., “open wound”, “orthopedic follow-up care”) were excluded from further analysis.

### Data processing and visualization

All data processing, statistical analyses, and visualizations were performed using the R programming language (Version 4.3). The analyses relied on a suite of packages from the Tidyverse ecosystem, primarily dplyr and tidyr for data manipulation and reshaping, ggplot2 for creating all graphical representations, and patchwork for combining multiple plots into composite figures.

### Data structure and preparation

The analysis was based on two main datasets: (1) annual counts and rates of non-unions and fractures from 2014 to 2023, and (2) aggregate counts and rates stratified by age. For the age-based analysis, data were categorized into ten distinct age classes: <1, 1–9, 10–17, 18–29, 30–39, 40–49, 50–59, 60–69, 70–79, and 80 + years. All datasets were further stratified by group: male, female, and total.

For quantitative modeling of the age distributions, each age class was represented by a single numerical value. We used the midpoint of each age interval for this purpose (e.g., 5 for the 1–9 age class, 24 for the 18–29 class). This is a standard approach for handling binned data, as it provides a simple and effective estimate of the central tendency for observations within each interval. This conversion allowed the application of statistical models designed for continuous data to the binned age group information.

### Statistical analysis

Two primary statistical methods were employed. First, to analyze temporal trends in non-union rates and absolute counts of fractures and non-unions, simple linear regression models were fitted using the lm() function. For each group, the regression slope, intercept, and the coefficient of determination (R²) were calculated to quantify the direction and strength of the linear trend over the ten-year period.

To describe the bimodal age distribution of fractures and non-unions, which likely reflect different etiological patterns (high-energy trauma in younger individuals and fragility-related fractures in older adults), a two-component Gaussian mixture model was fitted to the age-binned counts for each group. This statistical approach assumes that the observed distribution results from the combination of two underlying normal (Gaussian) distributions. Each component is characterized by its mean age (µ₁, µ₂) and standard deviation (σ₁, σ₂), while a mixing parameter (π) represents the relative contribution of each component to the overall distribution. The model parameters were estimated by maximizing the likelihood of the observed binned data. Using this approach, the overall age distribution can be decomposed into two underlying subpopulations.

The results were illustrated graphically using dual-axis plots to compare absolute amounts of fractures and non-unions, with regression lines and fitted mixture model curves overlaid on the observed data points.

## Results

During the study period, a total of 992,360 fractures and 20,268 non-unions of the foot and ankle were treated in German hospitals on an inpatient basis. Of the fractures, 443,878 (44.7%) occurred in males and 548,482 (55.3%) in females, corresponding to a male-to-female ratio of 1:1.23. Similarly, 9,005 (44.4%) non-unions were identified in males and 11,263 (55.6%) in females, yielding a sex distribution of 1:1.25 which was nearly identical to that observed for fractures. The mean non-union rate across all study years was 2.04%, with sex-specific rates of 2.03% in males and 2.05% in females, respectively.

### Temporal trend

**Fig. 1:**
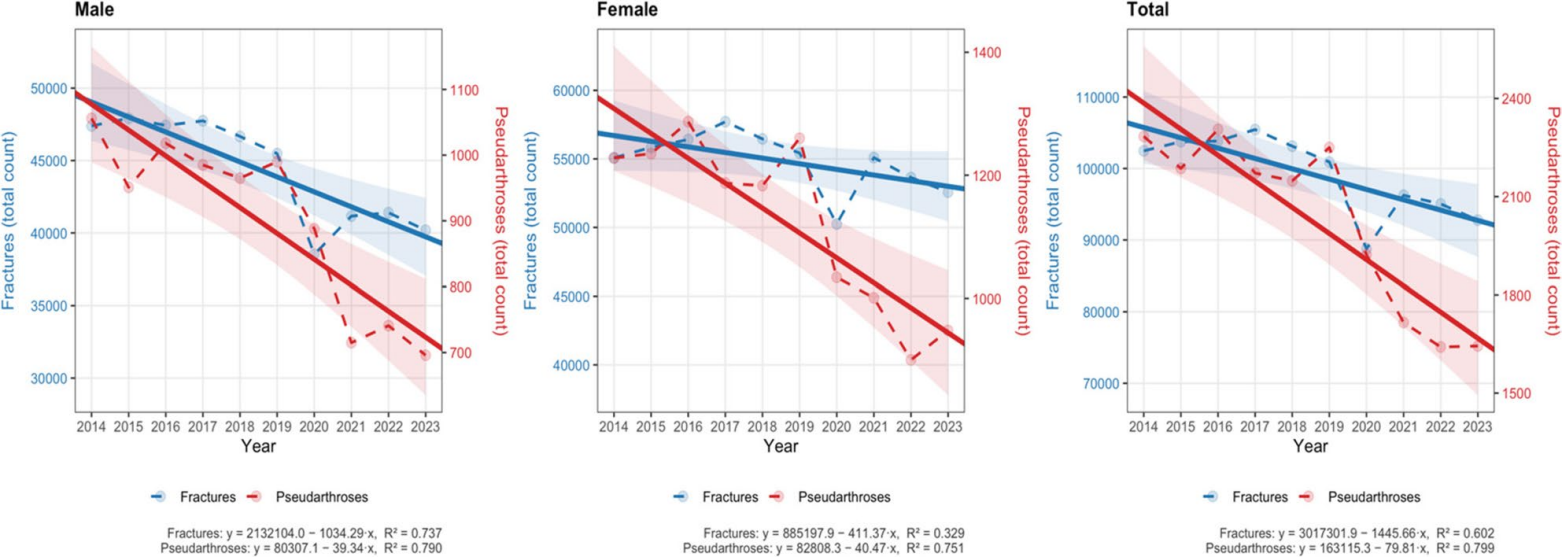
Temporal trends of fractures and pseudarthroses between 2014 and 2023, stratified by sex (male, female) and overall total. Each panel shows yearly case counts for Fractures (blue, left y-axis) and Pseudarthroses (red, right y-axis). Observed data are displayed as points connected by dashed lines, while fitted linear regression lines are overlaid as solid lines. Shaded ribbons represent the 95% confidence intervals of the regression models. Regression equations and coefficients of determination (R²) are reported in the figure captions for each panel

Between 2014 and 2023, the incidence of foot and ankle fractures requiring inpatient treatment demonstrated a substantial decreasing trend (Fig. 1). Case numbers remained relatively stable between 2014 and 2018, followed by a marked decline thereafter. An additional, distinct reduction was observed in 2020. Overall, fracture incidence declined by an average of 1,445 cases per year, corresponding to an annual relative reduction of 1.4–1.5%. Sex-stratified analysis revealed a more pronounced decline among males (2.2–2.6% per year) compared with females (0.7–0.8% per year).

**Fig. 2:**
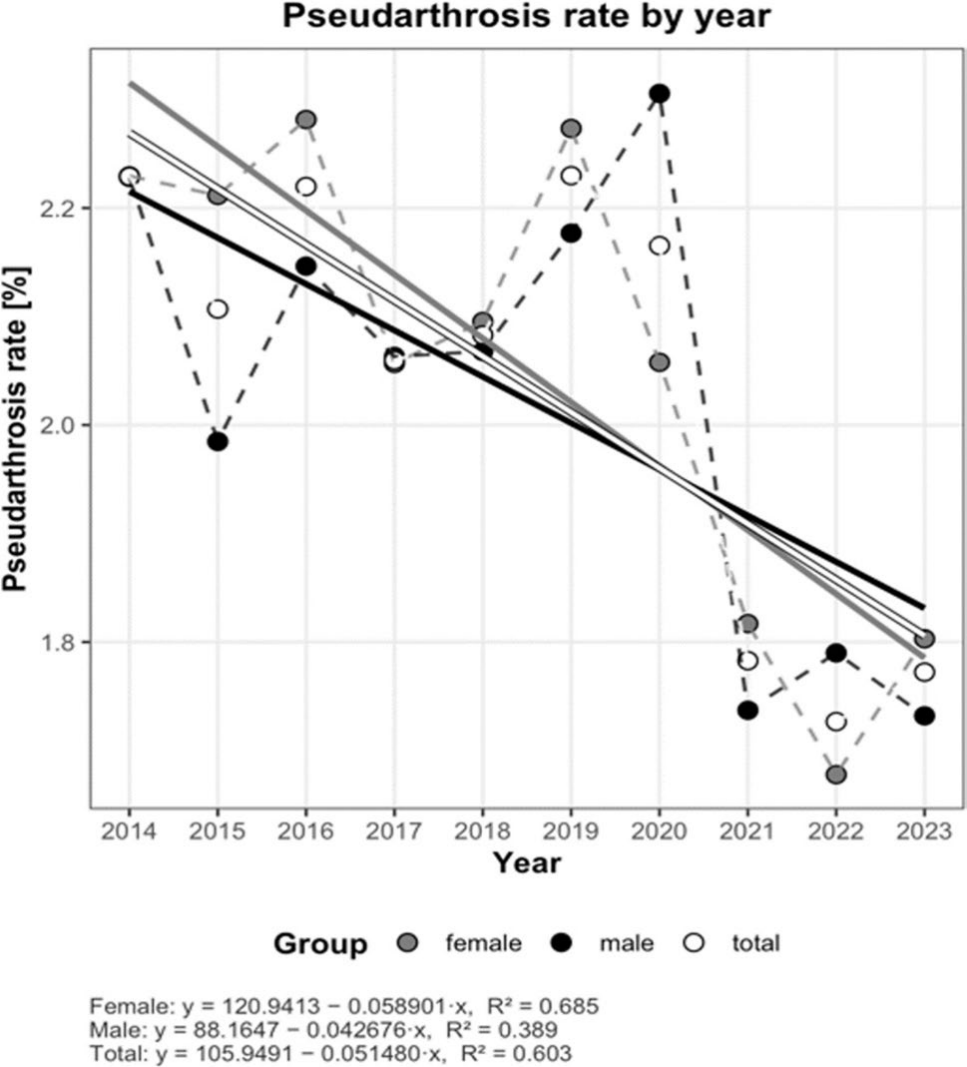
Pseudarthrosis rate by year (2014–2023). Scatter plots display the pseudarthrosis rate [%] for males (black), females (gray), and the total cohort (white) across the study period. Dashed lines connect the observed annual values. Straight regression lines (solid) are shown. Regression equations and coefficients of determination (R²) are reported below the panel

During the same period, the number of foot and ankle non-unions treated in inpatient care also showed a continuous decline (Fig. 1). The average annual reduction was 80 cases (3.5–4.9%), representing a steeper decrease than that observed for inpatient fractures. This decline was particularly pronounced among males, with an annual reduction of 3.7–5.6%, exceeding both the corresponding decline in females (3.1–4.4%) and the decrease in fractures within the same sex. This trend was also reflected in the non-union rate, which decreased by an average of 0.05% per year (Fig. 2).

### Age distribution

**Fig. 3:**
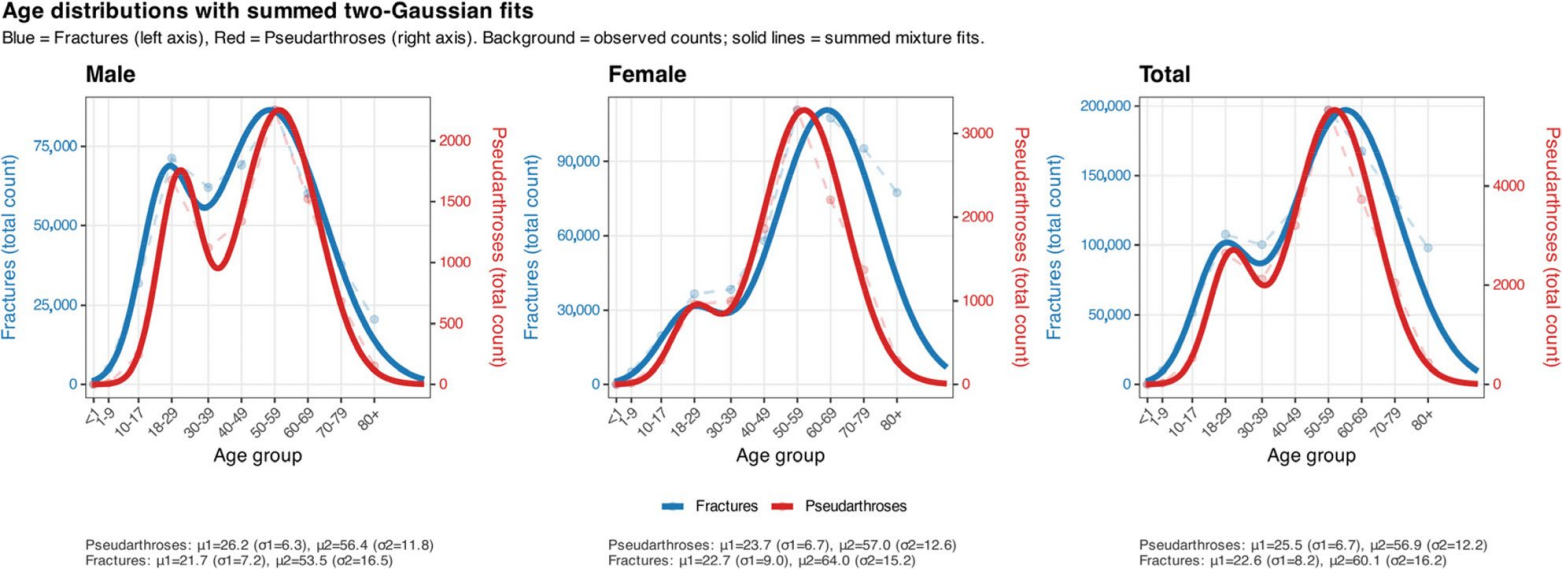
Age distributions of fractures and pseudarthroses (male, female, total). Each panel shows age-group counts for Fractures (blue, left y-axis) and Pseudarthroses (red, right y-axis). Observed data are displayed in the background as points connected by dashed lines; solid curves depict the sum of two Gaussian components fitted to the binned counts (mixture of two normals per outcome). Age groups are plotted at bin midpoints: <1 y = 0.5, 1–9 = 5, 10–17 = 14, 18–29 = 24, 30–39 = 35, 40–49 = 45, 50–59 = 55, 60–69 = 65, 70–79 = 75, and 80 + = 85 years. Mixture fits were estimated from binned data via maximum likelihood using CDF differences across bin edges (final bin 80–100 years); summed curves were scaled to the magnitude of observed counts, and pseudarthrosis curves were mapped to the left axis using a constant factor to enable dual-axis display. Reported means (µ) and standard deviations (σ) refer to the two fitted Gaussian components for each outcome and group

Both fractures and non-unions exhibited a bimodal age distribution, with a primary peak in the 50–59-year age group and a secondary peak among individuals aged 18–29 years (Fig. 3). This pattern was largely attributable to the bimodal distribution observed in males. In men, the highest proportion of fractures occurred between 50 and 59 years of age (19.5%), followed by the 18–29-year group (16.0%) and the 40–49-year group (15.6%). In contrast, the distribution in women was shifted toward older age groups, with the three largest proportions found in the 50–59-year (20.2%), 60–69-year (19.6%), and 70–79-year (17.4%) categories.

**Fig. 4:**
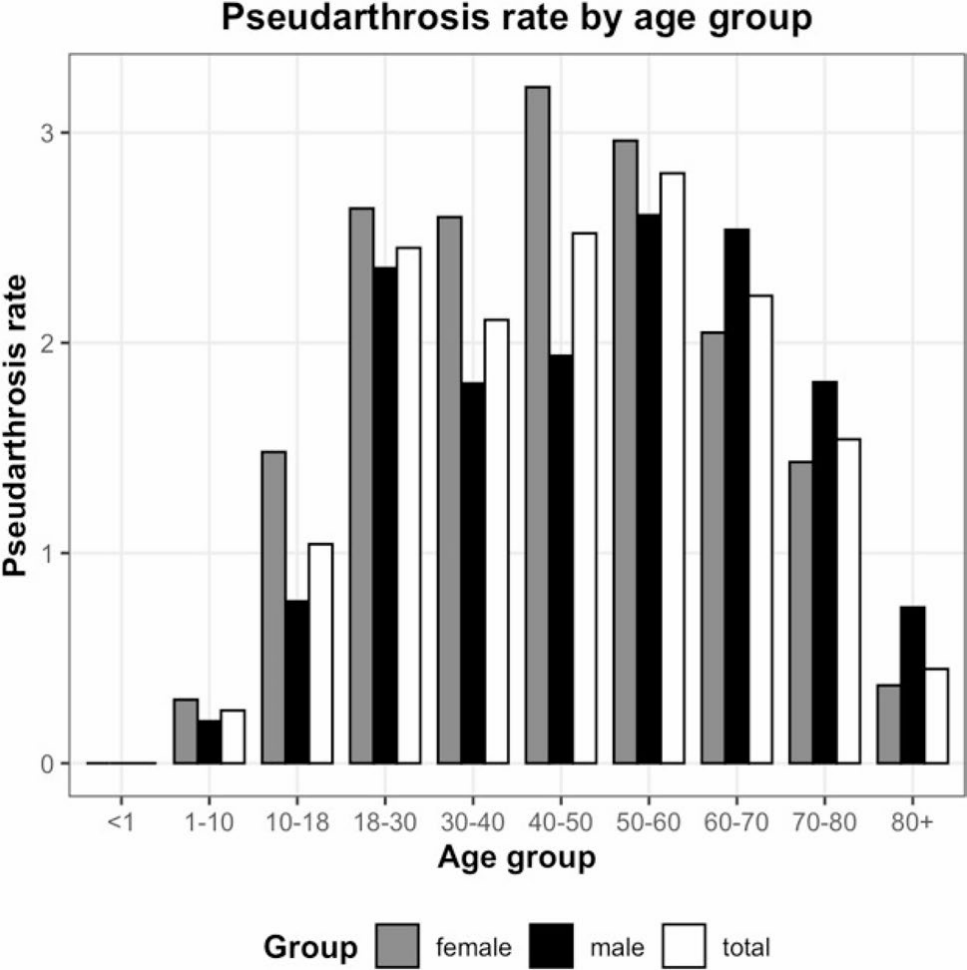
Non-union rate by age group. Bar plots show the proportion of non-unions in males (black), females (gray), and the total cohort (white) across predefined age classes. The rates are displayed as percentages relative to the total number of fractures within each age group

A similar pattern was observed for non-unions, although with a slight shift toward older patients in men (50–59 years: 25.0%; 18–29 years: 18.6%; 60–69 years: 16.9%) and toward younger patients in women (50–59 years: 29.1%; 60–69 years: 19.6%; 40–49 years: 16.5%). These distributions were reflected in pronounced age- and sex-specific differences in non-union rates. The highest overall non-union rate was observed in women aged 40–49 years (3.2%), followed by women aged 50–59 years (3.0%) and 18–29 years (2.6%). In men, non-union rates were consistently lower, with the highest values in the 50–59-year group (2.6%), followed by those aged 60–69 years (2.5%) and 18–29 years (2.4%). In both very young and very old patients, non-unions occurred below average and were thus comparatively rare (Fig. 4).

### Comorbidities

The ten most frequently coded secondary diagnoses during inpatient hospital stay are presented in Tables 1 and 2. Several comorbidities were clearly more prevalent among patients with non-union or delayed union compared with those with acute fractures. These included obesity (15.2% vs. 7.5%), allergy (8.0% vs. 4.3%), depressive episode (4.4% vs. not among the top ten), asthma (3.7% vs. not among the top ten), and sleep disorders (3.6% vs. not among the top ten). In contrast, atrial fibrillation (4.5%) and chronic kidney disease (4.1%) ranked among the most frequent comorbidities in patients with fractures but were not listed among the top ten in those with non-union.

**Table 1:** Ten most frequent comorbidities in patients with non-union/delayed union of the foot and ankle

Comorbidity	ICD-10	Count	Percentage (%)
Essential (primary) hypertension	I10	6372	31.4
Obesity	E66	3083	15.2
Other hypothyroidism	E03	2062	10.2
Type 2 diabetes mellitus	E11	1934	9.5
Personal history of allergy to drugs, medicaments or biological substances	Z88	1630	8.0
Disorders of lipoprotein metabolism and other lipidemias	E78	1376	6.8
Depressive episode	F32	892	4.4
Chronic ischemic heart disease	I25	808	4.0
Bronchial asthma	J45	750	3.7
Sleep disorders	G47	733	3.6

**Table 2:** Ten most frequent comorbidities in patients with fracture of the foot and ankle

Comorbidity	ICD-10	Count	Percentage (%)
Essential (primary) hypertension	I10	304,082	30.6
Type 2 diabetes mellitus	E11	89,782	9.0
Other hypothyroidism	E03	88,767	8.9
Obesity	E66	74,216	7.5
Disorders of lipoprotein metabolism and other lipidemias	E78	73,469	7.4
Chronic ischemic heart disease	I25	49,331	5.0
Atrial fibrillation	I48	44,907	4.5
Personal history of allergy to drugs, medicaments or biological substances	Z88	42,360	4.3
Chronic kidney disease	N18	40,289	4.1
Other functional bowel disorders	K59	36,074	3.6

## Discussion

In this study, nearly one million fractures and approximately 20,000 cases of non-union or delayed union of the foot and ankle were analysed over a ten-year period. The overall non-union rate was low, averaging around 2%, and was markedly lower than the 5–10% rates most frequently reported in the literature [7, 8, 10]. Age- and sex-specific differences were observed, with the highest non-union rate in women aged 40–49 years (3.2%) so that the study’s hypotheses could be confirmed.

As the analysis was limited to inpatient cases, while fractures of the foot and ankle are also managed in outpatient settings (both operatively and non-operatively), the actual number of fractures is likely higher. Nevertheless, because symptomatic non-unions are typically managed surgically and therefore treated in hospital, the present dataset is likely to provide a more accurate representation of their true incidence. It can therefore be assumed that the actual non-union rate may be even lower than reported here.

Comparable investigations reported a non-union rate of 2% [11] and 1.9% [9] across fractures of various anatomical sites, and only 1% for fractures of the foot and ankle [9]. However, this analysis included just 471 cases from the foot and ankle over a five-year period, representing a considerably smaller cohort than in the present study. Most other published work does not focus specifically on foot and ankle fractures and often includes sites with well-established higher non-union rates, such as the scaphoid or clavicle [7, 9].

While most studies report a higher incidence of non-union in men [5, 7, 9], the present analysis did not demonstrate sex-related differences in the overall non-union rate. When stratified by age, however, sex-specific patterns emerged. The highest non-union rates were observed in women aged 40–59 years and 18–29 years, whereas in men, rates peaked at older ages, particularly between 50 and 69 years, as well as in the 18–29-year group. At more advanced ages, non-union rates declined, indicating no positive linear association between age and the incidence of non-union. Similar findings have also been reported by Mills et al. [9]. Notably, the elevated rates observed among young adults warrant further attention. Potential contributing factors may include lifestyle influences or hormonal effects, particularly in women between 40 and 49 years of age. Moreover, in the very elderly, non-unions may be more frequently treated nonoperatively due to perioperative risk or pre-existing functional limitations, suggesting that the actual non-union rates in this population may be underestimated.

The age distribution of fractures of the foot and ankle demonstrated a characteristic pattern: biphasic in men, with peaks in young adulthood (18–29 years) and between 50 and 59 years, and monophasic in women, with the majority occurring at older ages. This distribution parallels the patterns described for fractures at other anatomical sites [9], and reflects the known sex-specific differences in etiology—occupational, sports-related, or high-energy trauma in men, and osteoporotic fractures in women.

Over the study period, a significant decline in the incidence of foot and ankle fractures was observed, particularly among men. An additional, distinct reduction was observed in 2020, most likely attributable to the impact of the COVID-19 pandemic. Similar findings have been reported from foot fractures in Sweden [12]. By contrast, Rupp et al. reported a marked increase in fracture incidence in Germany during years 2009–2019, but most notably for long bone fractures. Fractures of the malleolus lateralis, calcaneus, and malleolus medialis decreased by 6–20% [6]. In the context of evolving healthcare policy frameworks, surgical treatment of foot and ankle fractures is increasingly being performed in the outpatient setting. At the same time, conservative management strategies have regained greater attention. Both factors, alongside a true decline in fracture incidence, may have contributed to a reduction in the number of surgically and in the inpatient setting treated fractures. This uncertainty is inherent to the study design and cannot be entirely excluded.

Nevertheless, during the same period, the incidence of non-union decreased to an even greater extent than fracture incidence, resulting in a reduction in the non-union rate. This finding is encouraging, as it may reflect improvements in fracture care, such as the use of superior implants or optimized soft tissue management. Against the background of a potential increase in conservative fracture treatment, this trend remains positive and may further support the appropriateness of current therapeutic approaches.

In addition to age and sex, several risk factors are known to promote the development of non-union. These include patient-specific factors such as comorbidities, smoking, the use of certain medications, or nutritional deficiencies, as well as patient-independent risk factors such as fracture location and pattern, degree of displacement, severity of soft-tissue injury, infection, and type of fixation [3]. Comorbidities associated with an increased risk of non-union include obesity, diabetes mellitus, renal insufficiency, vitamin D deficiency, osteoporosis, alcohol abuse, and rheumatoid arthritis [7].

In the present study, analysis of coded secondary diagnoses revealed that obesity, allergies, depressive episodes, and sleep disorders were considerably more frequent among patients with non-union compared to those with fractures. The prevalence of obesity among non-union patients was 15.2%, which is lower than the overall prevalence of 19% in the German population, but higher than the prevalence among individuals aged 18–29 years (9.7%) [13], a group particularly affected by non-union. The prevalence rates of other comorbidities were broadly comparable to those observed in the general German population [14]. Nevertheless, the associations of comorbidities are descriptive, as causal relationships cannot be inferred due to the aggregated nature of the data. Therefore, no odds ratio was calculated for the development of a non-union.

This study has certain other limitations. As the dataset is limited to inpatient hospital cases, fractures managed conservatively or treated surgically on an outpatient basis could not be included. The same applies to non-union cases, although fewer of these are likely to be treated outside the inpatient setting, and thus presumably only a small proportion is missed. As the patient data is not linked longitudinally, multiple operations performed in the same patient may have been recorded as separate cases. However, more than 90% of patients with non-union can be successfully treated with the first surgery [15]. Due to the unlinked nature of the data, the reported non- union rates remain an estimate rather than a true per-fracture risk. Nevertheless, no more comprehensive data are currently available for Germany, and the large sample size makes this approach a robust approximation. Finally, the quality and completeness of diagnostic coding represent additional variables beyond the control of the study team.

## Conclusion

Despite the anatomical particularities of the so-called “last meadows,” non-unions following fractures of the foot and ankle remain rare and have declined steadily over the past decade. However, women between 40 and 59 years of age and young adults constitute groups at elevated risk, highlighting the importance of systematically addressing modifiable risk factors and tailoring preventive strategies to these populations.

## Data Availability

The datasets used and analysed during the current study are available from the corresponding author on reasonable request.
